# Characterization of the Complete Mitochondrial Genome of *Angulyagra polyzonata* and Its Phylogenetic Status in Viviparidae

**DOI:** 10.3390/ani15091284

**Published:** 2025-04-30

**Authors:** Shengjie Zhang, Kangqi Zhou, Xianhui Pan, Yong Lin, Jinxia Peng, Junqi Qin, Zhenlin Ke, Yaoquan Han, Zhong Chen, Xuesong Du, Wenhong Li, Pinyuan Wei, Dapeng Wang

**Affiliations:** 1 Guangxi Key Laboratory of Aquaculture Genetics and Breeding, Guangxi Academy of Fishery Sciences, Nanning 530021, China; shengjiefisher@outlook.com (S.Z.); zhoukqfisher@163.com (K.Z.); linnn2005@126.com (Y.L.); pengjinxia@hotmail.com (J.P.); hyqao@sina.com (Y.H.); cz1050@126.com (Z.C.); gslnlxr@163.com (X.D.); 2 College of Animal Science and Technology, Guangxi University, Nanning 530003, China; whli66@163.com; 3 College of Life Sciences, Southwest University, Chongqing 402460, China; 13022365884@163.com

**Keywords:** *Angulyagra polyzonata*, Viviparidae, mitochondrial genome, phylogenetic analysis

## Abstract

*Angulyagra polyzonata* is an economically important mollusk in China, but detailed insights into its mitochondrial genome remain scarce. In this study, we sequenced and comprehensively analyzed the structural features and selection pressures of the *A. polyzonata* mitochondrial genome. The maximum likelihood method and Bayesian phylogenetic inference method were used to construct a phylogenetic tree of *A. polyzonata* with 21 other species, including gastropods and bivalves. The full-length mitochondrial genome of 17,379 bp was found to include 22 transfer RNA genes, 2 ribosomal RNA genes, and 13 protein-coding genes, exhibiting similarity to the composition and arrangement of mitochondrial genes in other gastropod species. Notably, the Ka/Ks ratios of mitochondrial protein-coding genes (*nad5*, *cox3*, *nad3*, *nad2*, *cox1*, *cox2*, *atp8*, *atp6*, *nadl*, *nad6*, *cob*, *nad4l*, and *nad4*) were <1, which indicates that the snail genes of the three genera of the family may have been subjected to strong natural selection pressure during the evolutionary process, so that the number of synonymous mutations (ks) in genes was much more than that of nonsynonymous mutations (ka). Comparative genomic analysis indicated that, apart from the absence of *trnW* and *trnQ*, the gene composition of *A. polyzonata* shares a high degree of homology with other members of the conical snail family. Phylogenetic analysis demonstrated that the selected species could be classified into two primary clades in which *A. polyzonata* clustered with the Viviparidae family. This study bridges the knowledge gap regarding the mitochondrial genome of *A. polyzonata* and offers valuable insights into the systematic relationships within the Viviparidae family.

## Simple Summary

The full DNA sequence information (genome) of mitochondria is widely used in phylogenetic studies, which can provide scientific insights into the genetic evolution of species. In this study, we reported the complete mitochondrial genome of the mollusk species *Angulyagra polyzonata* and analyzed its characteristics in relation to its evolutionary degree and development. This analysis indicated that the gene arrangement and composition of mitochondrial genes of *A. polyzonata* are similar to those of other members of the Viviparidae family. Our study further showed that the species under investigation can be divided into two distinct branches (clades) in which *A. polyzonata* is located in close proximity to other Viviparidae snails. Collectively, we anticipate that our study’s findings will enhance the understanding of mitochondrial genome structure and contribute to the phylogenetic analysis of *A. polyzonata*.

## 1. Introduction

*Angulyagra polyzonata*, a species within the Viviparidae family of freshwater snails, is commonly found in rivers and ditches south of the Yangtze River in China, serving as an important ecological indicator species [1]. This snail acts as natural bait for poultry, fish, and crabs, and its attractive shell color is popular in the market, with annual consumption exceeding one million tons [2]. However, since 2012, habitat destruction, overfishing, and the invasion of another snail species, *Pomacea canaliculata*, have significantly reduced the distribution and abundance of *A. polyzonata*. Therefore, a clearer understanding of the phylogeny, taxonomy, and genetics of *A. polyzonata* is essential for its conservation and population restoration.

Globally, Viviparidae (Gray 1847) comprises 31 genera and 150 species, with no distribution in Antarctica or South America [3,4]. In China, there are nine genera and 70 species of Viviparidae, based on traditional morphological classification [5,6]. Recent studies of gastropod phylogeny indicate that molecular biological and traditional morphological methods yield conflicting results, with molecular data revealing some gastropod genera to be paraphyletic [7,8,9,10,11]. The availability of genetic data for molecular phylogenetic studies is essential for the conservation of biodiversity. *A. polyzonata* is one of the younger species of Viviparidae and the most abundant species in the natural distribution of *Angulyagra*. Research on the phylogeny of *A. polyzonata* may help to solve the problem of classification ambiguity in Viviparidae snails. Previous studies on *A. polyzonata* mainly focused on environmental ecological indicators [12], disease transmission [13,14], single-molecule marker genetic characterization [15], and habituation [16]. However, studies on the phylogenetic relationship of this species within the family are lacking. A reassessment of the phylogenetic relationships of *A. polyzonata*, incorporating more detailed gene structures and a wider scope, could yield more reference data for the conservation of wild population resources of *A. polyzonata*.

Mitochondria are essential organelles in eukaryotic cells, responsible for energy synthesis and the conversion of energy for life activities. With Nass and others [17] having discovered the existence of genetic material in mitochondria, successive research showed that all of the substances needed for replication, transcription, and protein translation are found in the organelle [18,19,20], demonstrating that the mitochondria house a relatively independent genetic transcription system [21]. A mitochondrial genome usually has three genetic characteristics: typical maternal inheritance; conservation of the coding region; and rapid evolution and high mutation rates of the control region and independent replication units [22]. Therefore, in addition to nuclear genes, mitochondrial DNA (mtDNA) is very useful in the study of molecular evolution.

In this study, we aimed to sequence the mitochondrial genome of *A. polyzonata*, analyze its structure, and compare it with the mitochondrial genomes of other confirmed snails in the same family. Additionally, we examined the evolutionary relationships of *A. polyzonata* within the Viviparidae family by reconstructing phylogenetic trees using whole mitochondrial genome sequences. This study presents the mitochondrial genome structure and phylogenetic relationships of *A. polyzonata* for the first time, providing valuable insights into the genetic evolution of Viviparidae snails.

## 2. Materials and Methods

### 2.1. Experimental Materials and DNA Extraction

**Figure 1:**
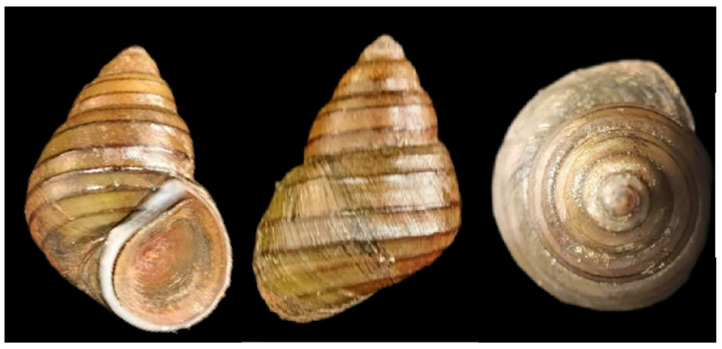
Photographs illustrating the morphology of *Angulyagra polyzonata*. Images from left to right show the front view, back view, and side view.

The chitinous shells of *A. polyzonata* are thick, robust, and conical in shape, and they feature seven dark brown spiral ribs on the body whorl. While the upper four ribs are prominent and protrude from the shell surface, the lower three are comparatively smaller and less noticeable (Figure 1). Snail specimens were collected from the Zhu Silkworm Reservoir (108°31′32.776″ E, 22°39′19.120″ N) in Xinjiang Town, Nanning City, Guangxi Zhuang Autonomous Region, China, and temporarily housed in a stepped water tank at the Guangxi Academy of Fishery Sciences. During this period, intermittent oxygen supply was provided to maintain optimal living conditions and allow for observation. Healthy, disease-free snails exhibiting strong activity were selected as experimental specimens. Genomic DNA extractions were performed using the Omega Animal DNA Extraction Kit, and the quality and integrity of the DNA samples were assessed using an Agilent 2100 Bioanalyzer(Agilent Technologies (China) Co., Ltd., Beijing, China, produced in Germany). The qualified library was sequenced and assembled by applying second-generation sequencing and third-generation sequencing platforms by Nanjing Genepioneer Technology Co., Ltd. (Nanjing, China). Sequencing was performed following the library for Illumina and the library protocol for Nanopore PromethION sequencing.

### 2.2. Mitochondrial Genome Sequencing and Assembly

Quality-verified DNA was fragmented by mechanical interruption (ultrasound), purified, and subjected to end repair to create blunt ends, followed by the addition of an adenine (A) to the 3′ end, ligation of a sequencing adapter, and fragment size selection by agarose gel electrophoresis. The sequencing library was then generated by PCR amplification. To obtain high-quality clean data, the raw data were filtered using fastp (v0.23.4; https://github.com/OpenGene/fastp, accessed on 15 December 2024) software [23]. Briefly, the sequencing adapter and primer sequences were trimmed, and reads with an average quality value < Q5 or an average mass value > 5 were removed. The mitochondrial genome was assembled using the mitochondrial genome sequence of the reference species, and the gene structure annotation of the assembled mitochondrial genome was used to generate the mitochondrial genome map. The assembly core module uses SPAdes (v3.10.1; https://github.com/ablab/spades, accessed on 15 December 2024) software [24] to assemble the mitochondrial genome. The next-generation sequencing data were aligned with the assembled mitochondrial sequences using MiniMAP2 (2.15-R905) and corrected with CANNU (Master-Snapshot) [25]; the genome size was set to 20 Kb, and the nanopore-raw mode was selected. LoRDEC (v0.6) software [26] was then used to compare the upper third-generation and second-generation data, followed by MUMmer (v4.0.0 beta2) to compare the corrected third-generation data to the assembly results, identify reads with overlaps > 300 bp that can bridge the gap, and subsequently connect the two ends of the gap. To obtain the final results, the assembly results were polished with second-generation data using Pilon (v1.23) [27] software.

### 2.3. Mitochondrial Genome Structure Annotation and Analysis

The Mitos2 (http://mitos2.bioinf.uni-leipzig.de, accessed on 15 December 2024) [28] mitochondrial online annotation tool was employed to annotate the assembled sequences using the following parameters: E-value exponent = 5; maximum overlap = 100; and non-coding RNA overlap = 100. The Mitos2 annotation results were compared with closely related species and manually corrected to obtain the final annotation results. The secondary structures of transfer RNAs (tRNAs) were derived from the annotation results and incorporated into the mitochondrial genome map using OGDRAW (https://chlorobox.mpimp-golm.mpg.de/OGDraw.html, accessed on 15 December 2024) [29].

Transposable element (TE)-derived repeats are different from tandem repeats and are distributed throughout the genome. Repeats were identified using Vmatch (v2.3.0; http://www.vmatch.de/, accessed on 15 December 2024) software [30] in combination with Perl scripts, with the following parameters: minimum length = 20 bp; hamming distance = 3; and four identification forms (forward, palindromic, reverse, and complement).

Relative synonymous codon usage (RSCU) is thought to be the result of a combination of natural selection, species mutation, and genetic drift, and it was calculated using a script written in Perl, as follows: (number of one of the codons encoding an amino acid/number of all codons encoding the amino acid)/(1/type of codon encoding the amino acid), and (actual frequency of use of the codon/theoretical frequency of use of the codon) [31].

In the genome, the leading strand is generally rich in G and T, while the lagging strand contains more A and C. The phenomenon of a shift in the base frequencies of A relative to T and C relative to G is called AT skew and GC skew, respectively [32]. The following formula is used to calculate mitochondrial genome skewness values: ATskew = (A − T)/(A + T); GCskew = (G − C)/(G + C). We used MAFFT (v7.310) software (https://mafft.cbrc.jp/alignment/software/, accessed on 15 December 2024) [33] to compare mitochondrial gene sequences between *A. polyzonata* and four species of snails in the family Viviparidae, and KaKs_Calculator (v2.0) software (https://sourceforge.net/projects/kakscalculator2/, accessed on 15 December 2024) [34] to calculate the KaKs values of genes.

### 2.4. Analysis of Taxonomic Family Development

Using *Anodonta lucida* and *Hyriopsis cumingii* as outgroups, evolutionary tree analysis was performed on protein-coding DNA sequences shared by 19 species of five families: Viviparidae, Hydrobiidae, Ampullaridae, *Semisulcospiridae*, and Unionidae (Table 1). Multiple sequence comparisons were performed using MAFFT v7.427 software (auto mode) for each coding sequence. The aligned coding sequences were concatenated end-to-end using RAxML (v8.2.10) software (https://cme.h-its.org/exelixis/software.html, accessed on 15 December 2024) [35], the GTRGAMMA model was selected, a rapid bootstrap analysis (bootstrap = 1000) was performed, and a maximum likelihood evolutionary tree was constructed. jModeltest2.1.10 (https://mybiosoftware.com/jmodeltest-phylogenetic-model-averaging.html, accessed on 15 December 2024) was used to calculate model parameters, and the optimal model was obtained as GTR + G [36]. The Bayesian phylogenetic tree was constructed by [37] (https://www.softpedia.com/get/Science-CAD/MrBayes.shtml, accessed on 15 December 2024), and Markov chain (MCMC) algorithm was used. It was run for a total of 2,000,000 generations, and samples were taken every 1000 generations. A total of 2000 trees were generated, 25% were discarded, and the remaining trees were used to calculate the posterior probability.

## 3. Results and Discussion

### 3.1. Mitochondrial Structural Characteristics

**Figure 2:**
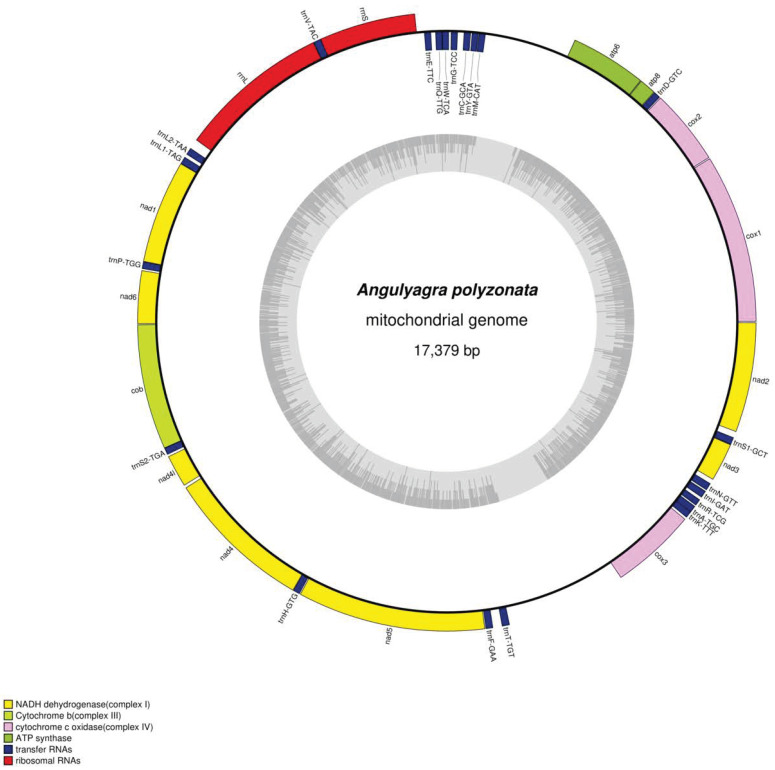
Ring map of the mitochondrial genome of *A. polyzonata* (PV083666). Genes encoded by the heavy (forward) strand are shown on outside the circle, and those encoded by the light (reverse) strand are shown on the inside of the circle. The inner gray circle represents the GC content.

The total DNA of A. polyzonata was sequenced, and the raw data were prepared for assembly, resulting in the second-generation sequencing data of 1.51 GB and the third-generation sequencing data of 1.5 GB (Supplementary Tables S1 and S2). We have determined that the complete mitochondrial genome of *A. polyzonata* (PV083666) has an average reading length of 17,379 bp (Figure 2), and it includes 22 tRNA genes, 2 ribosomal RNA (rRNA) genes, and 13 PCG genes. This genome is larger than the reported mitochondrial genomes of other freshwater snails, including *Planorbella pilsbryi* (13,720 bp) [36], *Hua aristarchorum* (15,691 bp) [38], *Cipangopaludina ampullacea* (16,892 bp) [39], *Tarebia granifera* (15,555 bp) [40], and *Cipangopaludina japonica* (16,995 bp) [41], confirming that Viviparidae snails have the longest mitochondrial genomes within the subclass Caenogastropoda [6].

**Figure 3:**
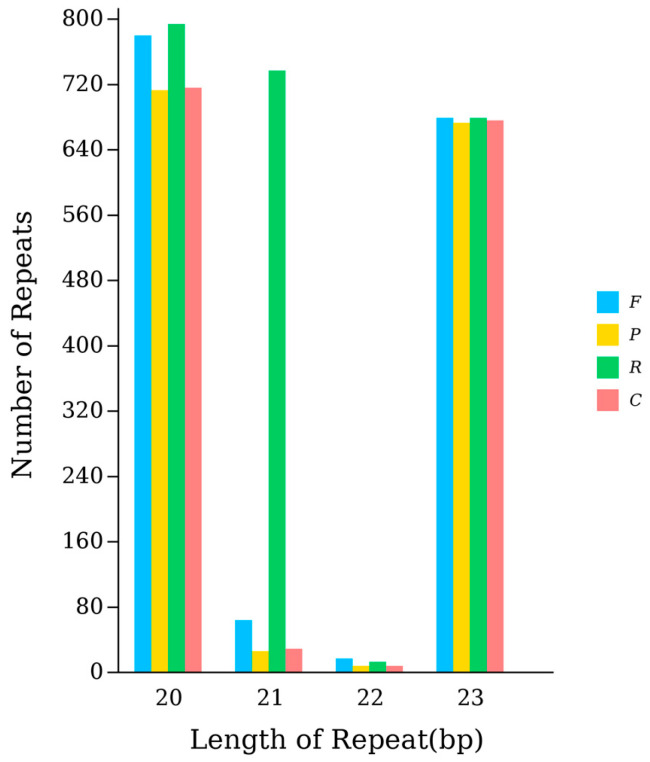
Statistical plot of scattered repeat sequences in the mitochondrial genome of *A. polyzonata*. *F*, forward repeat; *P*, palindromic repeat; *C*, complementary repeat; and *R*, inverted repeat.

Our data showed that seven of the tRNA genes (*trnE*, *trnQ*, *trnW*, *trnG*, *trnC*, *trnY*, and *trnM*) are encoded by the light strand (L-strand), while the remaining genes are encoded by the heavy strand (H-strand). Among the TEs of *A. polyzonata*, the numbers of repeated sequences with fragment lengths of 20 bp, 21 bp, and 23 bp were the highest (Figure 3). Studies have shown that TEs can affect genome size, directly or indirectly promote genomic rearrangement, and affect gene expression levels, rewriting gene regulatory networks [42]. Therefore, the large number of repetitive sequences in the *A. polyzonata* mitochondrial genome may have played a significant role in its evolution.

The *A. polyzonata* mitochondrial genome contains 31.24% A, 43.27% T, 16.04% G, 9.45% C, 74.51% A + T, and 25.49% G + C (Table 2), yielding a negative AT skewness value and a positive GC skewness value. A significant bias in the purine–pyrimidine ratio of the mitochondrial genome has been found in Viviparidae snails and other shellfish [43], and some scholars have suggested that it is a selective dependence of the species, although other evidence may be ambiguous due to the complexity of the problem [44,45]. We think it may also be related to the following reasons. The phenomenon of higher relative abundances of bases T and G in mtDNA may be related to mismatch repair [46]. During DNA replication, when double-stranded DNA is unwound into single-stranded DNA, mutations occur in response to environmental factors, activating the mismatch repair mechanism. In mismatch repair, there are higher probabilities of G-T mismatch and A-C mismatch, resulting in the presence of more G and T in single-stranded DNA. This may also be related to the deamination of C to uracil [47], which has a higher probability of hydrolysis in single-stranded DNA. If not corrected, it may also lead to a relative increase in the content of G and T. The control region is a critical area for mtDNA replication and transcription. Because of its low selection pressure and fast rate of evolution, it is rich in polymorphisms, making it a powerful tool for studying genetic diversity in organisms [48].

### 3.2. PCGs in the A. polyzonata Mitochondrial Genome

**Figure 4:**
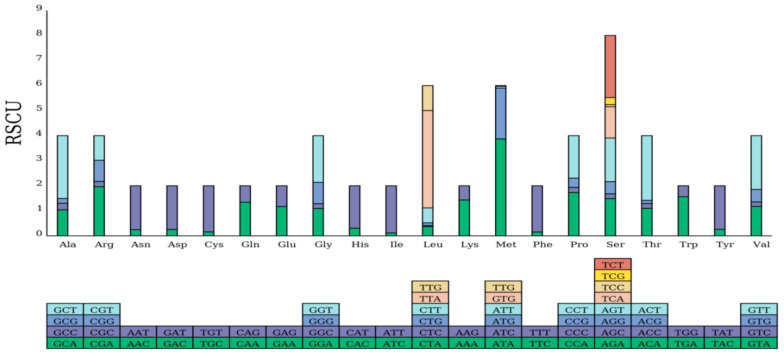
Relative synonymous codon usage (RSCU) of the mitochondrial genome of *A. polyzonata* (PV083666). The bottom graphic shows all of the sense codons used for each amino acid, with the height of each column representing the sum of the RSCU values of all the codons.

The PCGs of *A. polyzonata* mitochondria account for 64.00% of the total genes (Table 3). While most of these PCGs start with the canonical ATG codon, *nad6* and *nad5* start with ATT, and *nad4* starts with TGG. These findings indicated that, as the starting codon for transcription and translation, ATG has the highest efficiency and is preferred [49]. The RSCU and codon numbers of PCGs are listed in Table 4 and illustrated in Figure 4. Among the PCGs are 11 genes with standard stop codons, while two genes share non-standard, incomplete stop codons (T--). The appearance of incomplete stop codons is attributed to the presence of many scattered repeats at the 3′ end of these tRNA gene sequences [50]. Incomplete stop codons, which are generally very common in metazoans [51,52,53], can be converted into complete stop codons through post-transcriptional polyadenylation processes [54]. Differences in codon composition, especially regarding the phenomenon of incomplete termination codons, may provide insights into biological evolution.

In this study, three overlapping regions were identified between *trnW* and *trnQ*; *rrnS* and *trnV*; and *trnA* and *trnR*, with the *rrnS*–*trnV* overlapping region being the largest at 6 bp. Such overlapping regions can lead to certain differences in mitochondrial genome length among closely related species [51]. In the protein-coding region, a total of 3709 codons were used. On the basis of codon degeneracy, we know that serine is encoded by 8 codons, leucine and methionine are each encoded by 6 codons, and the remaining amino acids are encoded by 2–4 codons. The most common-sense codons are UUA (leucine), UUU (phenylalanine), AUU (isoleucine), GUU (valine), and AUA (methionine), with UUA and AUA having the highest RSCU values (>3.87), followed by ACU (threonine) and GCU (alanine), with RSCU values > 2.50. PCGs tend to use A or U in the third position of sense codons, but there are significant differences in codon usage among different species, especially those with distant genetic relationships. This is related to gene expression levels and structure, nucleotide composition, codon position within the gene, gene conversion and mutation bias, and environmental factors [55,56,57,58]. However, we found that the PCGs of the *A. polyzonata* mitochondrial genome possess non-coding regions at both ends; therefore, they were not included in the annotation, which specifically identifies functional elements such as the displacement loop (D-loop).

### 3.3. Comparative Analysis of Viviparidae Mitochondrial Genomes

**Figure 5:**
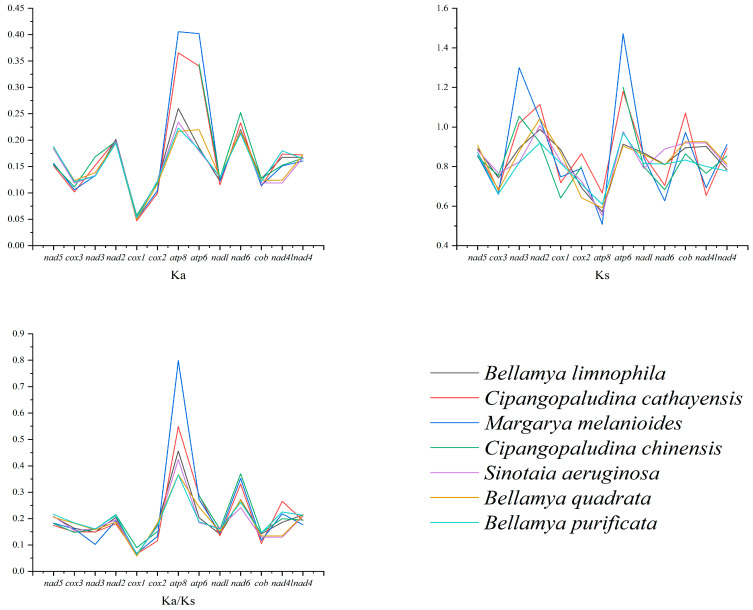
Nonsynonymous replacement rate (Ka), synonymous replacement rate (Ks), and Ka/Ks values of PCGs of *A. polyzonata* and other Viviparidae species. The PCG *atp8* of *C. chinensis* is poorly matched with the *atp8* of other species.

Next, we analyzed the evolutionary pressures faced by different species of Viviparidae by calculating the ratios of nonsynonymous replacement rate (Ka) to synonymous replacement rate (Ks) of mitochondrial PCGs. The results showed that the average Ka values of mitochondrial PCGs of seven snail species were not significantly different (0.1517–0.1790), with the exception of *atp8*, which had a higher average Ka value, indicative of a higher degree of positive gene selection among the species (Figure 5). The average Ks values of these genes also did not significantly differ among the seven species (0.8013–0.8786); however, the average Ks value of *atp6* was higher than that of the others, indicating that the protein-coding gene was found to have more synonymous substitutions at the same base position. Recent research has shown that Ka/Ks values can be used to determine whether there is selective pressure acting on PCGs [59], enabling the analysis of interspecies evolution rates within Viviparidae. The Ka/Ks values of all PCGs in the snails compared in this analysis were <1, indicating that purification selection dominated the evolution of these snails. Purification selection is a form of natural selection that tends to preserve genes essential for an organism’s survival and reproduction, maintaining efficient and stable function of genes by reducing the frequency of harmful mutations [60]. When genes undergo purification selection, nonsynonymous mutations are generally rejected by natural selection, while synonymous mutations occur relatively freely [61]. It can be seen that the evolution of these Viviparidae genes is mainly limited by function, and harmful mutations are selectively excluded, while neutral or beneficial mutations are retained. Among the PCGs, *cox1* (0.00674) evolved under strong purification selection, while *atp8* (0.4931), *nad6* (0.2995), and *atp6* (0.2382) evolved under relatively relaxed positive selection. Notably, the high Ka/Ks value of 0.7982 for *atp8* in *M. melanoides* led us to conclude that there is a strong positive selection trend in the evolution of this PCG. These broad evolutionary selection patterns also exist in aquatic animals, such as fish [62] and shrimp [63].

**Figure 6:**
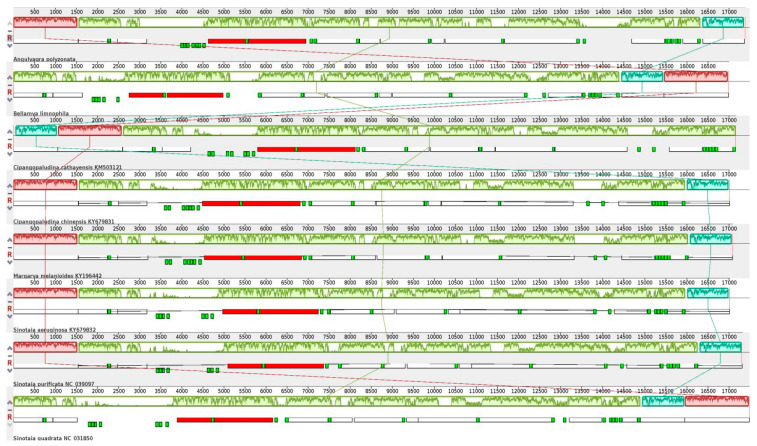
Comparison of the mitochondrial genomes of eight Viviparidae species, including *A. polyzonata*. Large rectangles represent sequence similarities between genomes; lines connecting rectangles indicate a collinear relationship; and smaller, shorter rectangles represent the location of the genes in each genome, with white used to indicate PCGs, green to indicate tRNA genes, and red to indicate rRNA genes.

Our comparison of the *A. polyzonata* mitochondrial genome sequence with the mitochondrial genome sequences of seven other species of Viviparidae revealed that, except for the deletion of tRNA genes *trnW* and *trnQ* in *Bellamya limnophila*, the mitochondrial genome lengths of the eight snails are similar, exhibiting three collinear relationships (Figure 6). However, there are variations in the length and position of some PCGs and RNA genes, indicating that these snails have both homologous and divergent genomic features. This result, to some extent, confirms the existing genus classifications of Viviparidae species *A. polyzonata as Angulyagra*; *Bellamya limnophila*, *Sinotaia aeruginosa*, *Bellamya purifica,* and *Bellamya quadrata* as *Bellamya*; *Cipangopaludina chinensis* and *Cipangopaludina cathayensis* as *Cipangopaludina*; and *Margarya melanoides* as *Margarya*.

### 3.4. rRNA and tRNA Genes in the A. polyzonata Mitochondrial Genome

**Figure 7:**
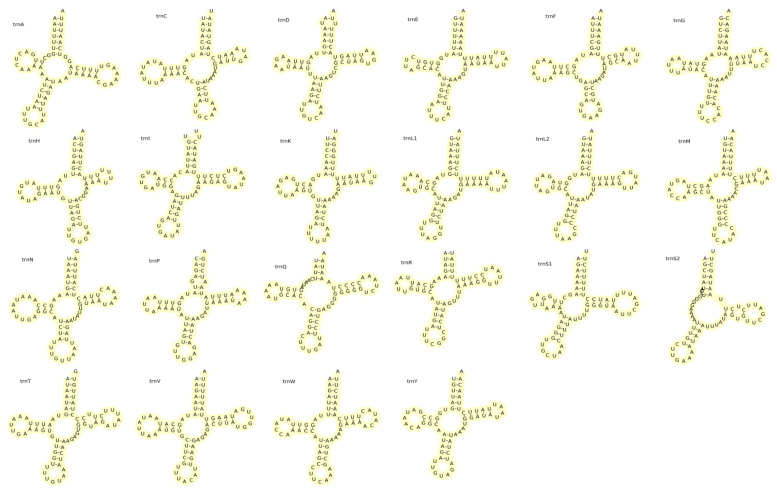
Secondary structures of the 22 mitochondrial tRNAs encoded by *A. polyzonata*.

The total length of rRNAs in *A. polyzonata* is 2260 bp (881 bp of short rRNA and 1379 bp of long rRNA), with an AT skewness of −0.025 and a GC skewness of 0.337. These rRNAs are encoded between *trnE* and *trnL*, with *trnV* as the dividing line (Table 3), consistent with some previously reported gastropod snails [64,65]. The 22 tRNAs encoded by the mitochondrial genome of *A. polyzonata* have a total length of 1484 bp, an AT skewness of −0.026, and a GC skewness of 0.219. Among them, 15 are encoded on the H-strand and seven 7 encoded on the L-strand. The secondary structures of the mitochondrially encoded tRNAs are shown in Figure 7. Except for *trnS2*, which lacks a dihydrouridine (DHU) stem, the other tRNAs all have a standard clover shape. The phenomenon of DHU stem deletion has also been found in mitochondrial tRNA studies of fish [66,67] and some crustaceans [68,69,70]. In addition, we observed 41 G:U pairings, one A:C pairing, and one U:C pairing in the tRNA of *A. polyzonata*, which often occur in DNA and RNA [71]. We noted that the high frequency of G:U mismatch in tRNA of *A. polyzonata* mitochondria indicates that it serves as a substitute for Watson–Crick base pairing, but also has important biological functions. The phenomenon of base mismatches in tRNA is beneficial for the recognition of tRNA by associated synthases, and also facilitates the formation of RNA higher-order structures and stable base pairing, making important contributions to RNA coding [72].

### 3.5. Phylogenetic Relationship

**Figure 8:**
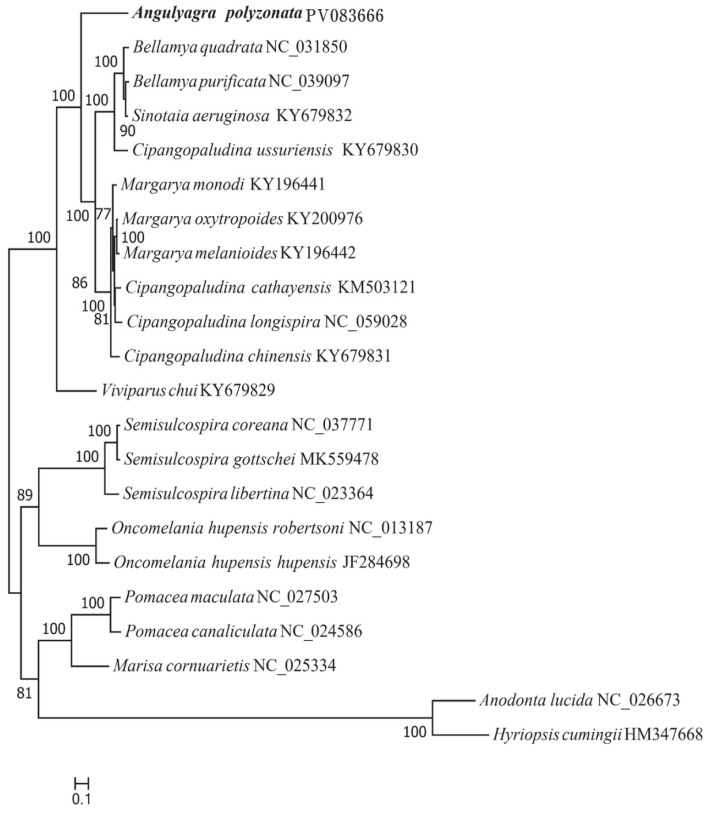
Phylogenetic tree constructed from the sequences of 13 PCGs in the mitochondrial genome of *A. polyzonata* (maximum likelihood estimation, MLE).

**Figure 9:**
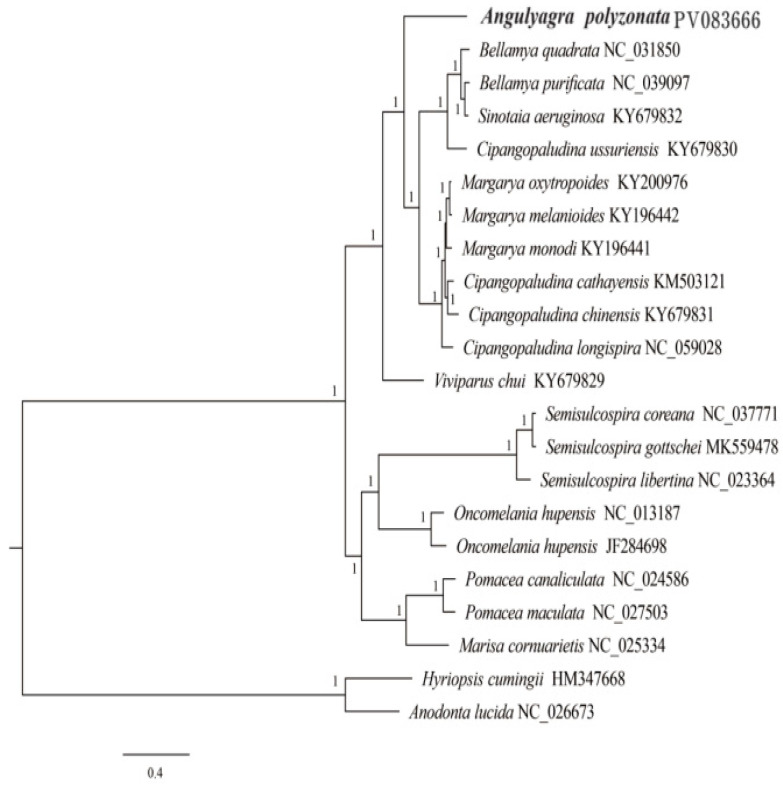
Phylogenetic tree constructed from the sequences of 13 PCGs in the mitochondrial genome of *A. polyzonata* (Bayesian Inference, BI).

A phylogenetic tree constructed using complete mitochondrial genome sequences of *A. polyzonata* and 21 mollusks (Figure 8 and Figure 9) clearly illustrated that, except for two bivalve outgroups of Unionidae (consistent with previous researchers’ understanding of the classification of these two genera) [6,39], the rest can be divided into two main branches, with Viviparidae as one branch, and Ampullariide, Hydrobiidae, and *Semisulcospiridae* as a second branch.

In Viviparidae, *Viviparis chui* belongs to *Cipangopaludina*, but it has a relatively distant phylogenetic relationship with the other species of Viviparidae, thus raises our concerns about the rationality and accuracy of snail classification among different genera of Viviparidae. We also observed that *Margarya* and *Cipangopaludina* are sister lineages. Interestingly, although *C. ussuriensis* belongs to *Cipangopaludina*, it is a sister lineage to several snails in *Bellamya*. Previous scholars’ studies on the mitochondrial genome of the *Cipangopaludina* snail have shown that some snails of this genus are grouped in the same category as *Margarya* or *Bellamya* [6,39]. This result is consistent with the conclusion of species classification status based on phenotype, and the same situation was also found in this study. However, Lu et al. [73] proposed that, despite the differences between *C. ussuriensis* and other species, it should still be classified as *Cipangopaludina*. Therefore, we believe that when conducting phylogenetic analysis using the mitochondrial genome as a method, it is necessary to combine multiple genomes and use multi-gene (such as ITS and 28S rRNA), multi-omics (whole-genome SNP), or morphological means to verify the results. This will also be an extremely important research direction in the future. The other branch is composed of *Semisulcospira*, *Oncomelania* (Gredler), *Pomacea*, and *Marisa*. While our findings clearly showed that *A. polyzonata* belongs to Viviparidae, it has not formed very close sister relationships with other Viviparidae snails, also affirming the rationality of its classification as *Angulyagra*.

## 4. Conclusions

This experiment presents the first comprehensive analysis of the mitochondrial genome of *A. polyzonata*, focusing on its structural characteristics through the study of 13 protein-coding genes and phylogenetic analysis. The mitochondrial genome of A. polyzonata is 17,379 bp in length, similar to other gastropod snails. Except for nad6, nad5, and nad4, which start with ATT and TGG, respectively, most PCGs start with the regular codon ATG, and there are two PCGs that share the irregular termination codon T--. Purification selection dominates the evolutionary process of 13 PCGs, and mitochondrial genome sequences show certain homology and differences compared to Viviparidae snail sequences. Except for the absence of DHU stem in trnS2, all other tRNAs have a standard clover shape. Phylogenetic analysis shows that although A. polyzonata also belongs to Viviparidae, it has not formed a sister relationship with other snails in the same family. However, we also found that the taxonomic status of some Viviparidae snails is still in a vague state, which will be an important direction for us to continue our efforts in the future.

## Figures and Tables

**Table 1:** Taxonomic information and GenBank entry numbers for all species used in the phylogenetic analysis.

Family	Genus	Species	Accession Number
Unionidae	*Anodonta*	*Anodonta lucida*	NC_026673
	*Hyriopsis*	*Hyriopsis cumingii*	HM347668
Ampullariidae	*Pomacea*	*Pomacea maculata*	NC_027503
		*Pomacea canaliculata*	NC_024586
	*Marisa*	*Marisa cornuarietis*	NC_025334
*Semisulcospiridae*	*Semisulcospira*	*Semisulcospira coreana*	NC_037771
		*Semisulcospira gottschei*	MK559478
		*Semisulcospira libertina*	NC 023364
Hydrobiidae	*Oncomelania Gredler*	*Oncomelania hupensis robertsoni*	NC_013187
		*Oncomelania hupensis hupensis*	JF284698
Viviparidae	*Bellamya*	*Bellamya quadrata*	NC_031850
		*Bellamya purificata*	NC _039097
		*Sinotaia aeruginosa*	KY679832
	*Cipangopaludina*	*Cipangopaludina ussuriensis*	KY679830
		*Cipangopaludina cathayensis*	KM503121
		*Cipangopaludina longispira*	NC_059028
		*Cipangopaludina chinensis*	KY679831
		*Viviparus chui*	KY679829
	*Margarya*	*Margarya monodi*	KY196441
		*Margarya oxytropoides*	KY200976
		*Margarya melanioides*	KY196442
	*Angulyagra*	*Angulyagra polyzonata*	PV083666

**Table 2:** Nucleotide composition and skewness of genes encoded by the heavy and light strands of the mitochondrial genome of *A. polyzonata*.

*Angulyagra_polyzonata*	Size (bp)	A%	T%	G%	C%	A + T%	G + C%	AT-Skew	GC-Skew
Mitogenome	17,379	31.24	43.27	16.04	9.45	74.51	25.49	−0.161	0.259
PCGs	11,123	26.95	44.40	17.96	10.69	71.35	28.65	−0.245	0.254
tRNAs	1484	35.85	37.74	16.11	10.30	73.59	26.41	−0.026	0.219
rRNAs	2260	36.06	37.92	17.39	8.63	73.98	26.02	−0.025	0.337

PCCs, protein-coding genes; tRNAs, transfer RNAs; rRNAs, ribosomal RNAs.

**Table 3:** Overview of the complete mitochondrial genome of *A. polyzonata*.

Gene	Position				Intergenic Length	Codon	
	Stand	From	To	Size		Start	Stop
*cox1*	H	1	1536	1536	0	ATG	TAA
*cox2*	H	1545	2237	693	8	ATG	TAA
trnD-GTC	H	2244	2308	65	6	/	/
*atp8*	H	2311	2466	156	2	ATG	TAA
*atp6*	H	2469	3164	696	2	ATG	TAA
trnM-CAT	L	3973	4038	66	808	/	/
trnY-GTA	L	4041	4104	64	2	/	/
trnC-GCA	L	4114	4181	68	9	/	/
trnG-TCC	L	4240	4305	66	58	/	/
trnW-TCA	L	4324	4390	67	18	/	/
trnQ-TTG	L	4389	4454	66	−2	/	/
trnE-TTC	L	4496	4561	66	41	/	/
rrnS	H	4631	5511	881	69	/	/
trnV-TAC	H	5506	5577	72	−6	/	/
rrnL	H	5578	6956	1379	0	/	/
trnL2-TAA	H	7044	7110	67	87	/	/
trnL1-TAG	H	7139	7206	68	28	/	/
*nad1*	H	7207	8148	942	0	ATG	TAA
trnP-TGG	H	8149	8214	66	0	/	/
*nad6*	H	8228	8710	483	13	ATT	TAG
*cob*	H	8716	9853	1138	5	ATG	T--
trnS2-TGA	H	9854	9917	64	0	/	/
*nad4l*	H	9936	10,232	297	18	ATG	TAA
*nad4*	H	10,256	11,596	1341	23	TGG	TAA
trnH-GTG	H	11,598	11,661	64	1	/	/
*nad5*	H	11,668	13,377	1710	6	ATT	TAA
trnF-GAA	H	13,383	13,450	68	5	/	/
trnT-TGT	H	13,531	13,601	71	80	/	/
*cox3*	H	14,687	15,466	780	1085	ATG	TAA
trnK-TTT	H	15,485	15,549	65	18	/	/
trnA-TGC	H	15,553	15,622	70	3	/	/
trnR-TCG	H	15,622	15,684	63	−1	/	/
trnI-GAT	H	15,709	15,776	68	24	/	/
trnN-GTT	H	15,788	15,857	70	11	/	/
*nad3*	H	15,898	16,249	352	40	ATG	T--
trnS1-GCT	H	16,250	16,318	69	0	/	/
*nad2*	H	16,373	17,371	999	54	ATT	TAA

**Table 4:** Relative synonymous codon usage and codon number of *A. polyzonata* mitochondrial PCGs.

Codon	No.	RSCU	Codon	No.	RSCU	Codon	No.	RSCU
UAA()	12	1.8462	AAG(K)	25	0.5682	CGG(R)	12	0.842
UAG()	1	0.1538	CUA(L)	34	0.3684	CGU(R)	14	0.9824
GCA(A)	43	1.036	CUC(L)	4	0.0432	AGA(S)	70	1.4856
GCC(A)	11	0.2652	CUG(L)	10	0.1086	AGC(S)	9	0.1912
GCG(A)	8	0.1928	CUU(L)	55	0.5958	AGG(S)	23	0.488
GCU(A)	104	2.506	UUA(L)	359	3.888	AGU(S)	82	1.74
UGC(C)	5	0.1588	UUG(L)	92	0.9966	UCA(S)	59	1.252
UGU(C)	58	1.8412	AUA(M)	151	3.8718	UCC(S)	4	0.0848
GAC(D)	10	0.2564	AUC(M)	0	0	UCG(S)	13	0.276
GAU(D)	68	1.7436	AUG(M)	79	2.0256	UCU(S)	117	2.4824
GAA(E)	52	1.1686	AUU(M)	3	0.0768	ACA(T)	34	1.0968
GAG(E)	37	0.8314	GUG(M)	0	0	ACC(T)	6	0.1936
UUC(F)	27	0.1552	UUG(M)	1	0.0258	ACG(T)	4	0.1292
UUU(F)	321	1.8448	AAC(N)	16	0.25	ACU(T)	80	2.5808
GGA(G)	68	1.0924	AAU(N)	112	1.75	GUA(V)	90	1.1728
GGC(G)	12	0.1928	CCA(P)	51	1.7288	GUC(V)	14	0.1824
GGG(G)	53	0.8516	CCC(P)	6	0.2032	GUG(V)	38	0.4952
GGU(G)	116	1.8636	CCG(P)	11	0.3728	GUU(V)	165	2.15
CAC(H)	11	0.3056	CCU(P)	50	1.6948	UGA(W)	83	1.566
CAU(H)	61	1.6944	CAA(Q)	41	1.3442	UGG(W)	23	0.434
AUC(I)	19	0.1172	CAG(Q)	20	0.6558	UAC(Y)	20	0.2614
AUU(I)	305	1.8828	CGA(R)	28	1.9648	UAU(Y)	133	1.7386
AAA(K)	63	1.4318	CGC(R)	3	0.2104	/	/	/

## Data Availability

The datasets presented in this study were submitted to the National Center for Biotechnology Information (NCBI) database.

## Supplementary Materials

The following supporting information can be downloaded at https://kdocs.cn/l/cmcE05oPhj0W. Table S1: Statistical table of second-generation sequencing data; Table S2: Statistical table of third-generation sequencing data; Table S3: Nonsynonymous substitution rate of mitochondrial genes in Viviparidae species; Table S4: Synonymous substitution rate of mitochondrial genes in Viviparidae species; Table S5: The ratio of the number of nonsynonymous substitutions per nonsynonymous site (Ka) to the number of synonymous substitutions per synonymous site (Ks) of mitochondrial genes in Viviparidae species.
